# The Immune Inductive Role of Hepatic Arterial Infusion Chemotherapy Prior to Atezolizumab Plus Bevacizumab Combination Therapy in Hepatocellular Carcinoma

**DOI:** 10.1016/j.gastha.2024.01.013

**Published:** 2024-02-01

**Authors:** Hiroyuki Suzuki, Miwa Sakai, Hideki Iwamoto, Shigeo Shimose, Takashi Niizeki, Masahito Nakano, Tomotake Shirono, Yu Noda, Etsuko Moriyama, Ryoko Kuromatsu, Hironori Koga, Takumi Kawaguchi

**Affiliations:** 1 Division of Gastroenterology, Department of Medicine, Kurume University School of Medicine, Kurume, Japan; 2 Liver Cancer Research Division, Research Center for Innovative Cancer Therapy, Kurume University, Kurume, Japan; 3 Iwamoto Internal Medicine Clinic, Kitakyushu, Japan

Atezolizumab and bevacizumab (Atez/Bev), a combination of immune checkpoint inhibitor and molecular targeted agent, has become the first-line therapy for patients with advanced hepatocellular carcinoma (HCC) and has improved outcomes.^1^^,^^2^ In the context of immunotherapy, treatment that follows the concept of the cancer-immunity cycle is important. Step 1 of the cancer-immunity cycle (release of cancer cell antigens upon cancer cell death) is essential for effective priming and effector phases.^3^ However, a therapeutic strategy for the efficient induction of step 1 of the cancer-immunity cycle has not yet been established. Hepatic arterial infusion chemotherapy (HAIC) and other locoregional therapies cause tumor necrosis and apoptosis, which may boost the antitumor effect of combination immunotherapy by promoting step 1.^4^, ^5^, ^6^ This study investigated the additive effect of HAIC as a neoadjuvant therapy for Atez/Bev therapy.

A total of 145 patients with advanced HCC treated with Atez/Bev at our institute between November 2020 and September 2022 were retrospectively enrolled in this study. The patients were divided into 2 groups: those who received New cisplatin plus 5-fluorouracil (FP) therapy, one of the regimens of HAIC using cisplatin (CDDP), 5-fluorouracil (5-FU), and lipiodol (specific therapy regimen is described in Supplementary Material and Methods)^6^ before Atez/Bev (New FP-Atez/Bev group: *n* = 27), and those who did not (Atez/Bev group: *n* = 118). We evaluated the therapeutic response using enhanced computed tomography according to modified Response Evaluation Criteria in Solid Tumors and calculated progression-free survival (PFS) and overall survival (OS) using the Kaplan-Meier method. Although not statistically significant, the New FP-Atez/Bev group was younger and had better liver function than the other group. (Table). Median PFS and OS were 9.2 and 18.4 months, respectively, with 39.9% objective response rate and 85.5% disease control rate (Figure A and B). The objective response rate in the New FP-Atez/Bev group was significantly higher than that in the Atez/Bev group (66.7% vs 40.7%, *P* = .014; Figure C). The median PFS and OS of the New FP-Atez/Bev group were significantly longer than those of the Atez/Bev group (Figure D). To understand the effect of New FP therapy on the tumor microenvironment (TME), we assessed 10 patients treated with New FP therapy before hepatic resection and 6 control patients without New FP therapy from another cohort. Immunohistochemistry for major histocompatibility complex class I (MHC-I), CD8, and programmed cell death ligand 1 (PD-L1) was performed and quantified as previously described.^7^^,^^8^ Compared to the control group, New FP therapy before hepatic resection significantly increased PD-L1-, MHC-I-, and CD8-positive cells in the TME (Figure E).

**Table:** Baseline Patients' Characteristics

Value	All, *n* = 145	New FP-Atez/Bev, *n* = 27	Atez/Bev, *n* = 118	*P*
Age (y)	72 [37–93]	70 [51–85]	73 [37–93]	.06
Sex, female/male	31/114	3/24	30/91	.11
Etiology, HBV/HCV/others	20/70/58	5/57/49	15/13/9	.15
Diabetes, −/+	81/64	18/9	63/55	.21
Body mass index	23.3 [15.5–35.3]	22.9 [17.7–30.8]	23.3 [15.5–35.3]	.99
Performance status, 0/1	116/29	24/3	92/26	.20
Child-Pugh class, A/B	139/6	24/3	115/3	.08
ALBI score	−2.40 [−3.50 to −1.34]	−2.39 [−3.10 to −1.34]	−2.40 [−3.50 to −1.44]	.20
mALBI grade, 1/2a/2b/3	43/50/51/1	7/11/8/1	36/39/43/0	.24
Neutrophil to lymphocyte ratio	2.54 [0.66–11.41]	2.76 [0.870–9.714]	2.70 [0.655–11.41]	.29
Maximum tumor diameter (mm)	33 [10–136]	44 [11–132]	32 [10–136]	.19
Alpha-fetoprotein (ng/mL)	41.25 [1.2–284,543]	79.9 [2–123,958]	38.1 [1.2–284,543]	.37
Des-γ-carboxy prothrombin (mAU/mL)	583 [11–3,637,900]	3610 [20–87,529]	423 [11–3,637,900]	.63

Data represent median [range].

ALBI, albumin-bilirubin; HBV, hepatitis B virus; HCV, hepatitis C virus.

**Figure:**
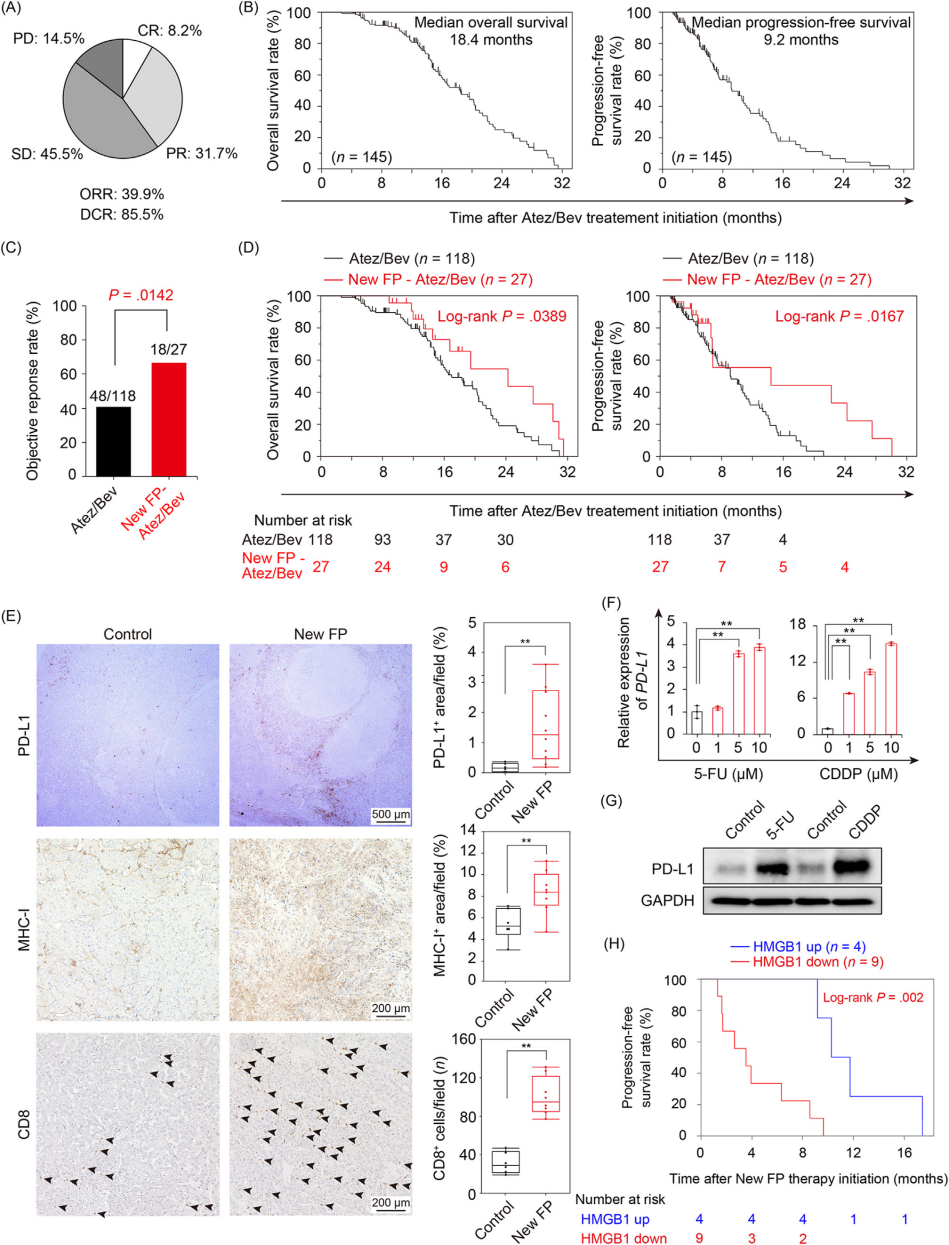
New FP therapy augments the effects of Atez/Bev therapy through immune induction in HCC. (A) Therapeutic assessment of Atez/Bev treatment. (B) Kaplan-Meier curves of the OS (left) and PFS (right) for all patients. (C) The objective response rates between the Atez/Bev and New-FP-Atez/Bev groups. (D) Kaplan-Meier curves of the OS (left) and PFS (right) between the Atez/Bev (*n* = 118) and New-FP-Atez/Bev (*n* = 27) groups. (E) Micrographs of immunohistochemistry (left panel) and quantification of these expressions (right panel) of PD-L1, MHC-I, and CD8 expression. Arrowheads represent CD8^+^ cells (∗∗*P* < .01). (F and G) The relative gene expression (F) and protein (G) of PD-L1 in HuH-7 cells stimulated by each concentration of 5-FU (left) or CDDP (right) for 24 h (∗∗*P* < .01). (G) Western blot analysis of 24 h of 10 uM of 5-FU or CDDP stimulation. (H) Kaplan-Meier curves of the PFS between the HMGB1 up (*n* = 4) and HMGB1 down (*n* = 9) groups. 5-FU, 5-fluorouracil; Atez/Bev, atezolizumab and bevacizumab; CDDP, cisplatin; CR, complete response; DCR, disease control rate; GAPDH, glyceraldehyde-3-phosphate dehydrogenase; HMGB1, high mobility group box 1; MHC-I, major histocompatibility complex class I; ORR, objective response rate; OS, overall survival; PD, progressive disease; PD-L1, programmed cell death ligand 1; PFS, progression-free survival; PR, partial response; SD, stable disease.

To examine the effects of CDDP and 5-FU, combination therapy using these drugs is commonly called FP therapy, on tumor cells, we performed reverse transcription-quantitative polymerase chain reaction and Western blotting for PD-L1 expression in HuH-7, a human HCC cell line. The primers and agents used in this study are described in the Supplementary Material and Methods. Interestingly, direct CDDP or 5-FU stimulation significantly increased PD-L1 expression in HuH-7 cells (Figure F and G).

According to Iwamoto et al, New FP therapy utilizing lipiodol possesses distinctive characteristics that differentiate it from other forms of FP therapy. Briefly, administration of a lipiodol suspension containing CDDP resulted in a mild vascular embolization effect and sustained release of CDDP. Additionally, continuous administration of 5-FU enhanced time-dependent antitumor effects. To confirm whether New FP therapy with these characteristics induced step 1 of the cancer-immunity cycle (release of cancer cell antigens upon cancer cell death), we evaluated serum high mobility group box 1 (HMGB1), one of the damage-associated molecular patterns,^9^ levels one month after New FP therapy. Of the 27 patients in the New FP-Atez/Bev group, serum HMGB1 levels could be measured in 13 patients. Patients with upregulated serum HMGB1 one month after New FP therapy (*n* = 9) had longer PFS than those with decreased (*n* = 4) (median PFS: 331 [range 276–524] vs 107 [40–290] days, *P* = .002) (Figure H).

If step 1 of the cancer-immunity cycle is not triggered, the antitumor effect of steps 2–7 of the cancer-immunity cycle (step 2, cancer antigen presentation; step 3, immune cell priming and T cell activation; step 4, immune cell migration; step 5, infiltration of immune cell into tumors; step 6, recognition of cancer cell by immune cell; step 7, killing of cancer cells) might be limited.^3^ Therefore, it is reasonable to implement locoregional therapy prior to immunotherapy including Atez/Bev, which aims to promote step 1. The study suggested that New FP therapy, a locoregional therapy with high local control potential, might have promoted step 1, as indicated by increased MHC-I-positive cells and serum HMGB1 and accelerated rotation of the cancer-immunity cycle, resulting in long PFS and OS.^6^ Moreover, New FP therapy might alter the TME into an immune hot state by infiltrating CD8^+^ T cells into the TME, and also the in vitro study showed that cytotoxic agents, such as CDDP and 5-FU, directly promoted the expression of PD-L1 in cancer cells. Consistent with a previous report, the degree of PD-L1 expression was correlated to the therapeutic response to immune checkpoint inhibitors.^10^ The current study had several limitations. First, the study design was retrospective, and the sample size was relatively small. Second, the observation period for the treatment was relatively short. Moreover, we could not assess the alternation of TME using the HCC tissue obtained before Atez/Bev initiation. However, the present study is the first to reveal the clinical significance of New FP as neoadjuvant therapy for Atez/Bev therapy. Thus, further accumulation of treatment experience and analyses in longer observation periods are needed to evaluate the true effects of Atez/Bev in real-world clinical practice.

In conclusion, the findings presented in the current study might offer important insights regarding the immune-inductive role of New FP therapy prior to Atez/Bev in HCC.
