# Supplementary material for: The Immune Inductive Role of Hepatic Arterial Infusion Chemotherapy Prior to Atezolizumab Plus Bevacizumab Combination Therapy in Hepatocellular Carcinoma
Source: Gastro Hep Adv. 2024 Feb 1;3(4):506–9. doi: 10.1016/j.gastha.2024.01.013 (PMC11307726; doi:10.1016/j.gastha.2024.01.013)
Supplement: Supplementary Materials [file mmc1.docx]

***Supplementary Materials***

**The immune inductive role of hepatic arterial infusion chemotherapy prior to atezolizumab plus bevacizumab combination therapy in hepatocellular carcinoma**

Hiroyuki Suzuki^1,2†^, Miwa Sakai^1†^, Hideki Iwamoto^1,2,3*^, Shigeo Shimose^1^, Takashi Niizeki^1^, Masahito Nakano^1^, Tomotake Shirono^1^, Yu Noda^1^, Etsuko Moriyama^1^, Ryoko Kuromatsu^1,2^, Hironori Koga^1,2^, and Takumi Kawaguchi^1^

^1^Division of Gastroenterology, Department of Medicine, Kurume University School of Medicine, Kurume, Japan

^2^Liver Cancer Research Division, Research Center for Innovative Cancer Therapy, Kurume University, Kurume, Japan

^3^Iwamoto Internal Medicine Clinic, Kitakyushu, Japan

†These authors contributed equally.

**Supplementary Material and Methods**

***The measurement of serum HMGB1***

Of the 27 patients in the New FP-Atez/Bev group, 15 were able to measure the serum high mobility group box 1 (HMGB1) levels using an HMGB1 ELISA Kit Exp (#326078738, Shino-Test Corporation, Tokyo, Japan).

***New FP therapy***

Initially, the patients undergoing angiography received fine-powder cisplatin (DDP-H, 50 mg/body; Nippon Kayaku Co. Ltd., Tokyo, Japan) suspended in 5–10 mL of lipiodol and 5-FU (250 mg bolus injection and 1250 mg continuous injection using an infusion balloon pump). The dose of the New FP regimen was determined by the treatment effect and adverse events.

***Immunohistochemical staining***

For quantification, CD8-positive cells (in 4–7 random fields per section) and MHC-I-positive areas (in 4–6 random fields per section) were counted at a magnification of ×200. All slides were examined using a confocal microscope (BZ-X700, Keyence Corporation, Osaka, Japan). Quantitative analyses were performed using Adobe Photoshop CC 2021 version 22.2.0.

***RT-qPCR and Western blotting for PD-L1 in HuH7***

HuH7, an HCC cell line, was purchased from the Japanese Collection of Research Bioresource Cell Bank (Tokyo, Japan). To evaluate the effects of CDDP and 5-FU, purchased from Tocris Bioscience, MN, USA, we performed reverse transcription-quantitative (RT-q) PCR and Western blotting. The cells were cultured under serum-starved conditions at 37°C overnight and then treated with the indicated concentrations of each drug or dissolved in dimethyl sulfoxide (DMSO) for 24 h. For RT-qPCR, primers (*GAPDH*, Hs02758991_g1; *CD274*, Hs00204257_m1) were purchased from TaqMan™, Applied Biosystems, CA, USA. Primary antibodies (PD-L1, #13684 and GAPDH, #sc-25778, Santa Cruz Biotechnology) were used for western blotting. Visualization of protein signals was achieved with the horseradish peroxidase-conjugated secondary antibodies (#NA934, 1:10,000, GE Healthcare Life Sciences, MA, USA).

***Statistical analyses***

All data are expressed as means ± standard error of the mean. Differences between two groups were examined for statistical significance using an unpaired Student’s *t*-test, and differences among multiple groups were examined using one-way analysis of variance, followed by a Fisher’s least significant difference test. *P* <.05 were considered statistically significant. Data analysis was performed using JMP Pro 17.0 software (JMP, Tokyo, Japan).
